# Micro- and Nanoplastics in Agricultural Crop Systems: From Environmental Particles to Plant Phenotypes and Food-System Relevance

**DOI:** 10.3390/plants15111594

**Published:** 2026-05-22

**Authors:** Muhammad Zubair, Abdul Karim, Maryam Noor, Laiba Bibi, Amina Qamar, Muhammad Ajmal Bashir, Muhammad Tanveer Akhtar

**Affiliations:** 1 College of Plant Protection, Yangzhou University, Yangzhou 225009, China; dh24024@stu.yzu.edu.cn; 2 College of Forestry, Jiangxi Agricultural University, Nanchang 330045, China; abdulk48034@gmail.com (A.K.); aminaqamar774@gmail.com (A.Q.); 3 College of Animal Science and Technology, Yangzhou University, Yangzhou 225009, China; maryamnoor1996@gmail.com; 4 Department of Botany, Centre for Plant Sciences and Biodiversity, University of Swat, Swat 19201, KP, Pakistan; laibakhan22@163.com; 5 Department of Agriculture and Forest Sciences (DAFNE), University of Tuscia, Via San Camillo De Lellis, s.n.c, 01100 Viterbo, Italy; 6 College of Horticulture and Landscape Architecture, Yangzhou University, Yangzhou 225009, China

**Keywords:** agroecosystems, plastic pollution, particle uptake, oxidative stress, crop contamination, crop quality

## Abstract

Micro- and nanoplastics (MPs/NPs) are increasingly recognized as persistent contaminants in agricultural systems, where repeated inputs from mulch films, biosolids, composts, irrigation water, and atmospheric deposition create sustained exposure pathways for crops. Although various studies report effects on crop growth and physiology, mechanistic interpretation remains limited because outcomes vary widely across experiments and are often discussed without appropriate attention to exposure context, particle properties, and evidentiary strength. This review advances an agroecosystem-centered, evidence-aware framework for interpreting how MPs/NPs influence crops from environmental entry to plant phenotype. We argue that crop responses cannot be inferred from polymer identity alone, but must be interpreted through the interacting effects of particle size, morphology, surface chemistry, weathering state, aggregation behavior, co-contaminant associations, and exposure matrix. Within this framework, crop responses are organized along a mechanistic chain linking environmental entry and plant contact, interface behavior at root and leaf surfaces, conditional barrier crossing and transport, ROS-centered stress signaling with hormonal and ionic regulation, and downstream effects on germination, root function, photosynthesis, biomass, productivity, and quality-related traits. Particular emphasis is placed on distinguishing surface association, supported internalization, and supported systemic translocation, because these categories carry distinct implications for edible-tissue occurrence, crop quality, and food-system relevance. Current evidence suggests that the soil–plant–food pathway is plausible and increasingly supported, but its interpretation remains constrained by uneven analytical rigor and limited field realism. Future progress will require realistic agricultural exposure designs, stronger polymer-specific confirmation, and closer integration of mechanistic evidence with agronomic and food-system endpoints.

## 1. Introduction

Microplastics (MPs conventionally defined as, <5 mm) and nanoplastics (NPs, often <1 μm) are progressively recognized as persistent contaminants across terrestrial, aquatic, and atmospheric environments, and their growing occurrence in agricultural systems has intensified concern over implications for soil functioning, crop performance, and food-system safety [1,2]. This concern is closely related to the sustained expansion of global plastic production, which reached approximately 436.7 Mt in 2022, while only about 9% was recycled [3]. As a result, a significant amount of plastic waste ends up in the environment, where physical fragmentation and chemical weathering generate progressively smaller particles that are more mobile, harder to remove, and more likely to interact with soil biota, plant surfaces, and co-occurring contaminants [4,5].

Agricultural and horticultural systems are particularly vulnerable because plastics are embedded in routine production practices. Mulch films, greenhouse coverings, irrigation-related materials, nursery containers, and plastic packaging cause repeated plastic loading. By the passage of time, these materials can weather, fragment, and become incorporated into soil [5,6]. In addition to direct use of plastic-based material, biosolids and other waste-derived amendments represent significant secondary inputs of MPs/NPs into agricultural soils. Field studies demonstrate that long-term biosolids application can lead to measurable microplastic accumulation in soil, while atmospheric deposition provides an additional and often underestimated source of contamination [7,8]. Agricultural soils should be seen not merely as passive recipients of plastic debris, but as active environmental reservoirs in which plastic particles are retained, transformed, and brought into prolonged interaction with soil biota and crops.

This agroecosystem perspective is crucial because crops occupy the functional boundary between environmental contamination and food production. Once MPs/NPs are present in crop environments, they can affect seed germination, root development, photosynthesis, oxidative balance, nutrient acquisition, and rhizosphere functioning [9,10,11]. Recent research further indicates that plastic contamination in agricultural soils may impair crop productivity and quality through combined effects on soil properties, microbial communities, contaminant behavior, and plant physiology, thereby extending the issue beyond conventional ecotoxicology into the broader domain of food security and agricultural sustainability [9,12]. Therefore, MPs and NPs are relevant in agriculture not just because of their persistence but also because of their ability to modify the soil-plant relationship that is essential to crop development.

Despite the expanding body of research, robust cross-system conclusions remain difficult because reported plant outcomes range from inhibitory to neutral and, in some cases, apparently stimulating. Much of this inconsistency arises because nominally similar exposures often represent biologically non-equivalent conditions. Outcomes can vary with particle size, polymer identity, surface chemistry, charge, shape, weathering state, and exposure matrix, meaning that broad labels such as polystyrene nanoplastics or microplastic exposure often conceal substantial mechanistic variation [13,14]. In these situations, it becomes challenging to determine whether an observed phenotype reflects true particle internalization, surface association, indirect soil-mediated stress, or combined particle-chemical effects. However, translation from controlled experiments to field-relevant crop inference remains limited because a substantial part of the experimental literature still relies on pristine or commercially produced particles, short-term seedling assays, hydroponic systems, and simplified single-particle exposures. These approaches are useful for mechanism-oriented studies, but they do not fully represent weathered, heterogeneous, and co-contaminant-associated particles in agricultural soils and irrigation-linked systems.

A second major source of uncertainty is the uptake and translocation of MPs/NPs in plants. Several studies report evidence consistent with the entry and mobility of nanoplastics under specific experimental conditions, including size- and property-dependent distribution patterns in plant tissues [13,14]. However, recent studies have found strong accumulation of micro- and nanoplastics at the root surface or root cap without evidence of uptake into internal tissues, highlighting the need to distinguish clearly between surface retention and confirmed internalization [15]. These inconsistencies should not be interpreted simply as biological variability; they also reflect methodological differences in particle labeling and decontamination procedures, matrix effects, detection limitations, and the use or lack of polymer-specific confirmation methods. Consequently, claims regarding uptake/absorption and transport require a more precise evidentiary framework than is frequently used in the current literature.

These concerns become even more essential when the discussion shifts from plant stress to crop quality, edible tissues, and human exposure. Studies of MPs/NPs in fruits, vegetables, and other edible plant materials have raised understandable concern. Still, the biological and food-safety significance of such findings depends on whether the particles are externally retained, surface-associated, or convincingly demonstrated within internal tissues. Recent studies confirm that micro- and nanoplastics may enter crop seedlings under controlled conditions and have been reported in edible produce, yet they also emphasize that many available studies still rely on standardized particles and laboratory conditions that do not accurately reflect actual agricultural exposure scenarios [16]. Therefore, the field requires analytical and conceptual frameworks that move beyond simple effect description and instead connect exposure route, particle properties, interface behavior, transport constraints, and downstream biological outcomes under agriculturally realistic conditions.

In this review, we propose an agroecosystem-centered, evidence-aware framework for analyzing micro- and nanoplastics phytotoxicity in agricultural crop systems. We concentrate on how exposure pathways and matrix conditions shape particle behavior at plant interfaces, how uptake and transport should be evaluated under precise analytical constraints, and how these processes translate into crop phenotypes through ROS, hormone, and ion-regulatory networks. We also evaluate the current evidence for translocation and the prevalence of edible tissue. By organizing the literature through this particles-to-phenotypes perspective, the review explains why similar exposures can produce different crop responses and provides a stronger basis for risk assessment and management in contaminated agricultural systems.

## 2. Scope, Definitions, and Evidence-Aware Vocabulary

This review does not treat microplastics (MPs) and nanoplastics (NPs) as a single, uniform category of plastic pollution. Instead, it treats them as particulate contaminants whose environmental behavior and biological significance depend on size, morphology, surface properties, weathering history, and exposure context. This distinction is particularly important in agricultural systems, where plastic inputs originate from various interconnected pathways, including soil amendments, irrigation water, atmospheric deposition, protected cultivation, and direct contact with roots, leaves, rhizosphere biota, and potentially edible tissues. In these situations, a precise vocabulary is necessary not only for comparing studies but also for judging whether reported plant responses are mechanistically meaningful and agriculturally relevant.

### 2.1. Operational Definitions and Size Classes Used in This Review

Definitions of MPs and NPs remain inconsistent across literature, particularly for nanoplastics. In this review, MPs are considered plastic particles smaller than 5 mm and greater than or equal to 1 μm, while NPs are defined as plastic particles approximately 1-μm in at least one dimension. This working division is consistent with commonly used environmental definitions and is also useful from a mechanistic perspective, because nanoscale particles are more likely to show colloidal behavior, matrix-sensitive aggregation, and conditional interactions with biological barriers, while larger microplastic fractions more often operate through surface retention, pore-scale interference, and soil-mediated effects [2,17]. As claimed by Gigault et al. (2018) [2], nanoplastics should not be viewed simply as very small plastics, but as particles within a size range that can behave colloidally, a crucial distinction in plant and soil systems since aggregation state can strongly alter effective exposure.

Even within these operational boundaries, exposure to NPs should not be treated as a single stable condition. Nanoplastics may aggregate into larger clusters depending on ionic strength, dissolved organic matter, weathering state, and surrounding matrix chemistry, which means that nominal particle size often does not predict actual behavior in soils, hydroponic solutions, irrigation water, or on leaf surfaces [18,19,20]. Therefore, explicit size-window descriptions such as 50 nm, 100–500 nm, or 1–10 μm are preferable to broad MPs/NPs labels when comparing experiments or interpreting mechanisms.

### 2.2. Particle Properties Required for Mechanistic Inference

A fundamental reason that plant studies often provide conflicting conclusions is that many experiments report only polymer identity and nominal mean size, while omitting the particle descriptors that determine environmental behavior and plant interactions. For mechanistic interpretation, at least five categories of information should be treated as minimum reporting requirements: size distribution, shape and morphology, surface charge or functionalization, aging or weathering state, and aggregation or corona behavior under the actual exposure conditions. Here, corona behavior refers to the formation of organic, mineral, microbial, or chemical coatings on particle surfaces in environmentally complex matrices, which can alter particle mobility, adhesion, and biological interactions.

Size distribution should be reported rather than average diameter alone, because a fine-particle fraction may dominate internalization potential, whereas larger fractions may dominate surface retention, pore blockage, and soil-structural effects [19,21]. Shape and morphology should also be specified, since spheres, fragments, fibers, and films differ in mobility, persistence, contact mechanics, and interaction with soil pores or plant [10,22]. Surface charge or functionalization is equally important because it strongly influences aggregation, adhesion to roots and leaves, and the intensity of physiological responses. Experimental evidence demonstrates charge-dependent differences in accumulation and phytotoxicity, indicating that surface chemistry is a core mechanistic variable rather than a minor methodological detail [23,24].

The aging or weathering state of particles also needs a precise definition. In agricultural environments, plastics rarely remain pristine; they are altered by ultraviolet radiation, oxidation, abrasion, wet-dry cycles, and repeated interaction with soil minerals and organic matter. These processes can change surface roughness, chemical reactivity, fragmentation behavior, and additive release, thereby modifying both environmental fate and biological response [12,18,25]. Aggregation state, corona formation, and co-contaminant association should also be specified wherever applicable, because they can shift an exposure from a nominal particle-only treatment toward a combined physical-chemical stress scenario. This is particularly crucial in agricultural soils, where MPs/NPs may interact with fertilizers, metals, pesticides, and dissolved organic matter, thereby influencing contaminant transport and bioavailability [26,27,28,29].

Without these descriptors, a study may still report only plant effects, but it cannot adequately support mechanistic inference or explain why outcomes differ across crops, environmental matrices, or contamination pathways. This level of reporting decides whether a result can be understood environmentally, rather than just as a simplified laboratory observation.

### 2.3. Exposure Routes Across Agroecosystems: Soil, Irrigation-Linked Water, Hydroponics, and Foliar Deposition

Mechanistic interpretation also depends on where particles initially come into contact with the plant and how the surrounding matrix modifies their behavior. In agricultural systems, soil exposure is the dominant route because repeated inputs from plastic mulches, composts, biosolids/sewage sludge, fertilizer-associated plastics, wastewater irrigation, and atmospheric deposition contribute to a persistent soil reservoir. Consequently, agricultural soils are increasingly recognized as major sinks for MPs/NPs [26,30,31,32]. Crop responses may result from both direct root contact and indirect soil-mediated pathways since MPs/NPs can change pore structure, aggregation, water dynamics, nutrient cycling, microbial activity, and co-contaminant mobility once they are in the soil [17,28,33,34,35].

This soil-centered perspective matters because it immediately connects contamination sources to crop-system consequences. Reviews of agricultural soils consistently emphasize that MPs can disrupt agroecosystem functioning, crop performance, food security, and potentially human health through their effects on soil biota, plant growth, and contaminant transport [36,37,38,39]. Therefore, plant studies that disregard source realism and the exposure matrix risk overinterpret mechanisms that might not translate well to agricultural areas.

Irrigation-linked exposure requires separate attention because it connects water contamination directly to agricultural soil and crop environments. MPs and NPs have been found to enter agricultural systems through reclaimed and wastewater-based irrigation, which can lead to repeated loading of the root zone and soil pore water, as well as, in certain cases, direct contact with aerial plant surfaces [40,41,42,43]. This pathway is especially relevant here because it integrates the soil and water dimensions of contamination within the same production system.

Hydroponic systems serve a different purpose. They are valuable for isolating physiological and molecular responses under controlled conditions, but they may also distort particle behavior because aggregation, sedimentation, and effective dose at the root surface can differ substantially from those in real soils or irrigation-affected fields [18]. Therefore, hydroponic evidence is informative for mechanism-oriented studies, but it should not be directly applied to agroecosystem risk without careful attention to matrix chemistry and environmental realism.

Foliar exposure has become increasingly relevant because atmospheric deposition of fine plastic particles can deliver MPs/NPs directly to crop canopies and edible surfaces. Recent plant studies acknowledge leaf-surface retention and, in certain conditions, stomatal entry as plausible processes, but they also show that foliar uptake claims are particularly prone to contamination artifacts and therefore require rigorous washing controls and polymer-specific confirmation [10,23,44]. This distinction is particularly crucial for food-system interpretation, because particles retained on the external surface of edible tissues are not equivalent to particles confirmed within internal tissues.

**Figure 1:**
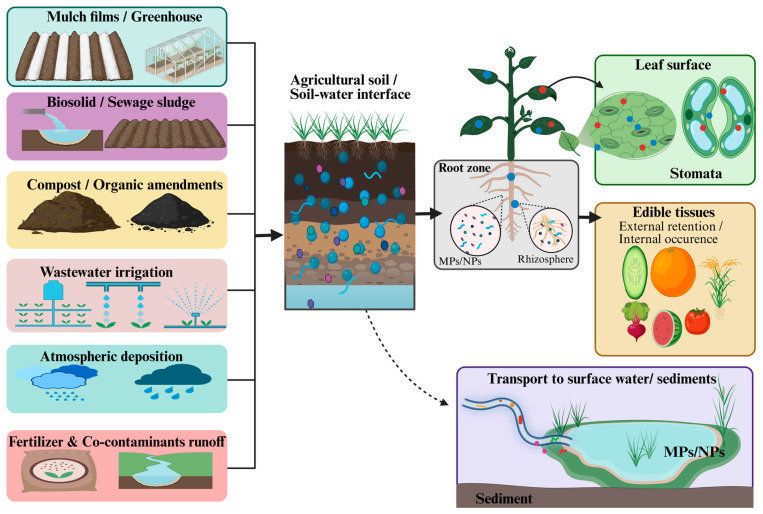
Source to crop exposure pathways of MPs/NPs in agroecosystems.

Exposure route should therefore be treated as a mechanistic determinant rather than a minor experimental detail. It shapes which plant interfaces are involved, which processes are plausible, and how strongly a given result informs the soil, water, crop, and food dimensions of agricultural microplastic contamination. The main agroecosystem exposure pathways and their likely crop relevance are summarized in Table 1 and conceptually illustrated in Figure 1.

### 2.4. Evidence-Aware Terminology for Plant Exposure and Transport

Several important concepts are employed here in a limited and evidence-aware manner due to the methodological heterogeneity of the literature. Surface association refers to particles retained on external plant surfaces, including root caps, epidermal interfaces, leaf cuticles, or stomatal regions, without evidence that they have crossed into internal tissues. Internalization is employed only when evidence supports particle presence within plant tissues beyond external surfaces. Translocation refers to movement from the initial contact site to distal tissues. In contrast, systemic translocation is reserved for cases in which internal localization in distal organs is supported by sufficiently rigorous detection and contamination control.

These distinctions are especially important in crop and food-quality research because accumulation on root surfaces or external edible tissues may occur without convincing evidence of uptake into internal tissues. Therefore, without orthogonal confirmation, claims about edible-tissue contamination, crop-quality consequences, or food-chain transfer should not be automatically inferred from exterior residues or from fluorescence-only detection processes.

The same principle applies to terms such as phytotoxicity, uptake, and mechanism. In this review, phytotoxicity is not treated as synonymous with any statistically significant plant response; rather, it refers to adverse biological outcomes or stress-associated reprogramming linked to particle exposure under defined conditions. Similarly, a mechanistic claim is not inferred from a single endpoint alone, but from a coherent linkage among exposure properties, plausible interface or transport processes, and downstream physiological, biochemical, or molecular outcomes.

## 3. From Environmental Particles to Crop Phenotypes: A Mechanistic Framework

A persistent limitation of the MPs/NPs literature is that studies often move directly from a nominal exposure label, such as polystyrene-nanoplastics (PS-NPs), to an endpoint such as reduced biomass, elevated ROS, or altered root growth, without precisely defining the causal steps in between. This tendency contributes substantially to the apparent inconsistency of the field. Similar exposure labels may indicate biologically non-equivalent conditions because particle size distribution, surface charge, morphology, aging state, aggregation behavior, and matrix chemistry can vary significantly across experiments, thereby altering whether the dominant process is surface retention, soil-mediated stress, conditional internalization, or combined particle-chemical toxicity [9,18,20]. This issue is amplified in agricultural systems since crops are not exposed through a single isolated matrix but rather through interconnected compartments such as soils, irrigation-linked water channels, composts, biosolids, mulch-derived residues, and atmospheric deposition.

**Figure 2:**
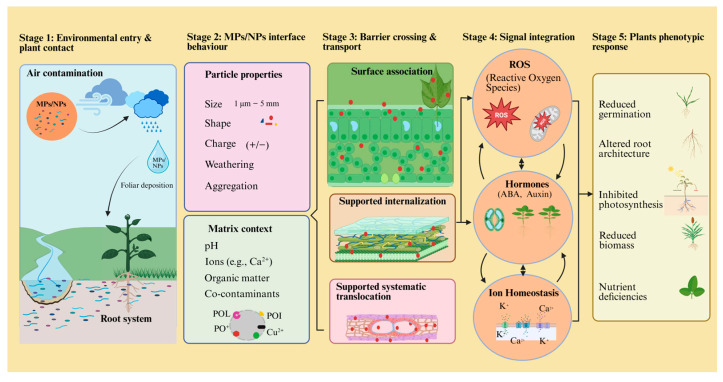
Five-stage particles-to-phenotypes framework linking environmental entry and plant contact, interface behavior at root and leaf surfaces, barrier crossing and within-plant transport, ROS-, hormone, and ion-regulatory signaling, and crop growth, productivity, and food-system outcomes.

To organize this complexity, this review adopts a particles-to-phenotypes framework that links five mechanistic stages: (i) environmental entry and plant contact, (ii) interface behavior shaped by particle properties and matrix context, (iii) barrier crossing and within-plant transport under explicit evidence constraints, (iv) signal integration through ROS-, hormone-, and ion-regulatory networks, and (v) phenotype execution in crop growth, physiology, productivity, and quality-related traits. This framework is meant to enhance interpretation rather than to impose a strict linear pathway, because the biological significance of exposure may emerge at different stages depending on the crop, the matrix, and the strength of the evidence [48,49]. It also provides a structure for explaining why some studies report clear inhibition, others weak or neutral effects, and others non-monotonic or context-dependent responses. The five-stage particles-to-phenotypes framework adopted in this review is summarized in Figure 2.

### 3.1. Environmental Entry and Plant Contact

The first stage deals with how MPs/NPs enter crop environments and where they first come into contact with the plant. In agroecosystems, this stage is shaped by a number of factors, including degradation of plastic mulches, biosolid and compost applications, reclaimed or wastewater-based irrigation, and atmospheric deposition [50,51,52,53]. These pathways determine more than the mere presence of particles. They also influence particle weathering history, associated chemicals, aging state, and spatial distribution at the point of plant contact. Plant exposure in agricultural landscapes is therefore better understood as a source-to-interface issue than as a simple dose-delivery event.

In soil-based systems, MPs/NPs typically have their first contact at the root-soil interface, where they interact with mucilage, exudates, pore water, minerals, and microbial communities [54,55,56]. Under these conditions, early plant responses may occur through indirect effects on the rhizosphere as much as through direct interaction with root surfaces. Irrigation-linked exposure modifies this contact stage by changing not only particle loading, but also the chemical and physical circumstances under which roots initially come into contact with MPs/NPs. Foliar exposure creates a distinct interface because atmospheric deposition can place fine plastic particles directly on crop canopies and edible surfaces, although leaf-surface retention, stomatal association, and confirmed internal uptake should not be treated as equivalent processes [16,23,44,57]. For this reason, the entry route already constrains which downstream mechanisms are plausible.

### 3.2. Interface Behavior as the First Mechanistic Filter

After environmental entry, the biological meaning of exposure is shaped by what occurs at the plant interface. This first mechanistic filter depends on effective size, morphology, surface chemistry, weathering state, aggregation, and matrix conditions, and it determines whether particles remain dispersed, accumulate at plant surfaces, alter local pore-scale environments, or act as carriers of contaminants [18,19,58,59].

### 3.3. Barrier Crossing and Within-Plant Transport Under Evidence Constraints

After particles interact with root or leaf surfaces, the next question is whether particles remain externally associated or progress toward internalization and transport within the plant. Proposed routes include apoplastic movement, crack entry, endocytosis-like uptake of smaller particles, and stomatal entry in foliar exposures, but uptake should not be treated as a default consequence of exposure [10,14,20,60]. Because the strength of transport claims depends heavily on analytical rigor, this issue is examined in more detail in Section 5.

### 3.4. Signal Integration and Phenotype Execution

Once MPs/NPs disturb the plant interface, with or without confirmed internalization, the biological consequences are mediated through regulatory networks rather than through a single isolated endpoint. Oxidative imbalance is a major response hub, while hormonal regulation and ion homeostasis shape how particle-associated stress is expressed as altered germination, root architecture, photosynthetic performance, biomass accumulation, and quality-related traits [9,61,62,63,64,65]. ROS should therefore be interpreted not only as a damage marker, but as a signal-processing node whose consequences depend on exposure intensity, duration, and plant buffering capacity.

Through this regulatory layer, upstream interactions are expressed as altered germination, root architecture, photosynthetic performance, biomass accumulation, nutrient status, and, under sustained exposure, quality-related traits. These execution outcomes are the point at which mechanistic interpretation becomes agronomically meaningful, because they determine whether exposure remains within a compensatory range of plant response or progresses toward measurable impairment of crop performance.

### 3.5. Why Similar Particles Can Produce Different Crop Responses

A central advantage of the present framework is that it helps explain why apparently similar particles do not always produce similar crop responses. Much of this variability reflects differences in effective particle behavior, environmental history, matrix context, and evidentiary strength. The following section focuses on the interface rules that generate much of this variation.

## 4. Interface Rules: Why Outcomes Vary Across Crop Systems

In this review, interface rules refer to the set of factors that govern particle behavior at plant surfaces before broader conclusions are drawn about transport, phytotoxicity, or food-system relevance. These rules help explain why some studies report pronounced inhibition of growth or photosynthesis, whereas others report weaker, neutral, or mixed effects under particles that appear similar in nominal description. They also clarify why responses observed in simplified laboratory systems do not always translate directly to agricultural fields.

### 4.1. Surface Retention and Microenvironmental Effects at Roots and Leaves

In many crop-relevant scenarios, the first biologically meaningful event is not immediate internalization, but particle retention at external surfaces. At the root-soil interface, MPs/NPs may accumulate within mucilage, around exudate-rich zones, on mineral surfaces, and within biologically active pore-water microenvironments. Even if particles do not enter internal tissues, they can alter root conditions by influencing pore continuity, water distribution, nutrient diffusion, contaminant mobility, and rhizosphere microbial activity. Under these conditions, physiological responses may result from changes in the local root environment rather than proven particle movement into the plant.

This distinction is important because many reported crop phenotypes may originate from microenvironmental disruption rather than from within-plant movement of particles. In soil-based systems, such effects are often amplified by matrix complexity, since roots do not interact with particles in isolation but with particles embedded in a dynamic physical and biochemical environment. This is one of the reasons why soil and agroecosystem-focused evaluations consistently advise that root-level responses should be regarded as part of a larger soil–plant system rather than as isolated toxicological occurrences.

Fine particles on aerial plant surfaces can accumulate on the cuticle, around stomatal openings, or in thin moisture films. These interactions can alter the duration of particle contact, local surface chemistry, and even gas exchange, even if clear evidence of internal uptake remains rare. This issue is especially relevant for crop canopies and edible organs, because external retention on leaves, fruits, or vegetables may still matter for contamination, handling, and exposure assessment, while remaining biologically distinct from confirmed within-tissue occurrence.

### 4.2. Aging, Weathering, and Additive Release as Modifiers of Plant Exposure

In an agricultural environment, plastic does not remain chemically or physically stable. In agricultural environments, mulch films, irrigation components, and other field plastics are exposed to sunlight, oxidation, abrasion, temperature fluctuations, and repeated wet–dry cycles. These weathering processes alter particle roughness, surface chemistry, fragmentation behavior, and additive release, thereby changing how MPs/NPs aggregate, adhere, and exchange chemicals at plant interfaces [53,58,66,67]. As a result, pristine laboratory particles should not automatically be treated as direct surrogates for field-derived crop exposure.

Additive release and degradation products add a further layer of complexity. In crop systems, MPs/NPs may function not only as physical particles but also as sources or carriers of reactive chemicals, especially after weathering [30,68,69,70]. Under such conditions, plant responses may reflect a combination of exposures, in which physical contact, oxidative stress, and associated chemicals all contribute to the outcome. This point is particularly important for agricultural interpretation because it connects interface behavior directly to broader concerns about crop quality, soil contamination, and food-system relevance.

### 4.3. Co-Exposure and Vector Effects: When Particles Amplify Other Stresses

In agricultural systems, MPs/NPs are rarely found in isolation. They coexist with fertilizers, metals, pesticides, antibiotics, dissolved organic matter, and plastic-associated chemicals, which means that crop exposure is often better understood as a co-exposure problem than as a single-contaminant problem. Recent agroecosystem studies emphasize that MPs can alter the availability, transport, and biological impact of other pollutants, thereby changing how roots encounter and respond to them [9,71]. Under these conditions, particles may act as stressors themselves, as well as vectors or amplifiers of other stresses.

This helps explain why similar crop phenotypes might evolve distinct interface mechanisms. A reduction in growth, photosynthesis, or nutrient uptake may reflect direct particle stress in one system, yet in another system, the same phenotype may result from a combined effect involving sorbed contaminants, altered nutrient dynamics, or matrix-driven changes in rhizosphere behavior. For agricultural interpretation, this distinction matters because it prevents crop responses from being attributed too narrowly to particle presence alone. Mechanistic statements are more compelling when they consider what else the particles are carrying, sorbing, or interacting with under field-relevant conditions. These vector effects also matter for food-production systems because MPs/NPs may modify the mobility and biological impact of other substances already present in soil or water.

## 5. Barrier Crossing and Transport: Boundary Conditions and Evidence Limits

Among the most contested questions in the MPs/NPs literature is whether particles that encounter crop surfaces can traverse biological barriers and translocate within the plant system. This issue is crucial for both mechanistic interpretation and the translation of results into claims about edible tissues, crop quality, and food-chain exposure. The most defensible conclusion at present is that uptake is neither categorically absent nor universally established. Rather, barrier crossing and transport should be conceptualized as conditional processes governed by particle physicochemical properties, plant anatomical architecture, exposure route, matrix conditions, and analytical rigor applied in detection and quantification.

In root-mediated exposure systems, the cell wall-apoplast continuum constitutes the primary physicochemical barrier governing particle translocation. Whether particles remain externally associated or move toward internal tissues is not determined by nominal diameter alone. It also depends on effective hydrodynamic size, aggregation dynamics, surface physicochemistry, and matrix-specific interactions. This mechanistic complexity accounts for the divergent outcomes reported across the literature, wherein certain investigations document findings consistent with localized cellular internalization, while others demonstrate preferential accumulation at root cap and epidermal interfaces without substantive evidence of apoplastic or symplastic entry into deeper root architecture [10,15,20]. From an agronomic and crop-safety perspective, this distinction carries considerable interpretive weight, as biologically consequential responses may manifest independently of confirmed systemic translocation, particularly when root–surface dynamics and rhizospheric microenvironmental conditions are substantively perturbed.

Multiple entry routes have been proposed in recent studies, encompassing crack entry at discontinuities associated with lateral root emergence, apoplastic movement through permissive interfaces, and endocytosis-like uptake for smaller particles. In foliar exposure systems, stomatal penetration has similarly been postulated for fine-fraction particles under select environmental conditions [20,60]. The theoretical plausibility of these pathways, however, does not constitute sufficient grounds for their uncritical assumption across heterogeneous exposure scenarios. Systematic reviews show that translocation claims are highly sensitive to methodological factors. These include particle labeling, washing and surface-decontamination procedures, matrix interference, detection limits, and polymer-specific confirmation methods [10,20]. Accordingly, surface association, experimentally substantiated internalization, and empirically confirmed systemic translocation must be rigorously maintained as epistemologically distinct evidentiary categories, rather than treated as operationally interchangeable descriptors within the interpretive framework.

**Figure 3:**
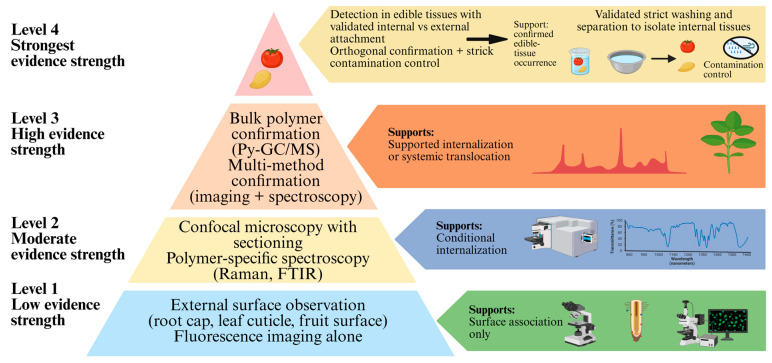
Evidence hierarchy for uptake, translocation, and edible-tissue claims.

This evidentiary distinction becomes particularly significant in foliar and edible-tissue investigations. Fine particles deposited onto leaf surfaces, fruit pericarps, or grain structures may remain externally retained on cuticular layers or within surface microarchitectures, and such residues carry fundamentally different biological and food-safety implications than particles convincingly demonstrated within internal tissue compartments. Consequently, claims regarding edible-tissue occurrence or food-chain relevance warrant evaluation against a more stringent analytical standard than claims about surface contact alone. The critical interpretive question is not merely whether an analytical signal was detected, but whether the experimental design can convincingly exclude confounding contributions from surface contamination, laboratory cross-contamination, fluorescent dye artifacts, and erroneous spatial misinterpretation of localization signals. Because uptake claims vary greatly in evidentiary strength, the distinction among surface association, supported internalization, supported translocation, and edible-tissue occurrence should be interpreted through an explicit evidence hierarchy (Table 2; Figure 3).

Strong uptake claims cannot be supported solely by low-level data, but they can help prove contact or suggestive localization. Although it is still limited in the absence of orthogonal confirmation, moderate-level evidence offers more reliable support for internal occurrence. High-level evidence is appropriate for stronger inference when contamination control and polymer-specific confirmation are both robust. Claims involving edible tissues require the strictest standard because external residues and internal occurrences do not carry the same biological, agronomic, or food-system meaning.

The hierarchy summarized in Table 2 is particularly important when studies extend their conclusions to edible tissues or food-system relevance. External residues on roots, leaves, fruits, or grains do not provide the same evidence as particles demonstrated within internal tissues, yet this distinction is often blurred in broader discussions of food-chain risk. Therefore, interpretations about crop contamination, dietary exposure, or consequences for food quality should be proportionate to the strength of the transport evidence and should not rely on single-method detection pipelines or fluorescence-only signals without orthogonal confirmation [14,16].

Additionally, it is critical to avoid exaggerating the contribution of transportation to the explanation of agricultural responses. Numerous significant consequences in agricultural systems could occur in the absence of verified internal particle mobility. Even when within-plant transport is still unknown, significant physiological and agronomic effects can be produced by altered soil structure, disturbed nutrient dynamics, rhizosphere stress, changing contaminant mobility, and persistent root–surface interactions. Therefore, the lack of compelling transport evidence should not be taken as proof of biological insignificance. Instead, it implies that the interface between plant surfaces and contaminated environmental matrices may be where MPs and NPs are most important in many crop systems.

When interpreted in this sense, barrier crossing is best understood as a limited and evidence-based phase in the broader particles-to-phenotypes pathway. The review is strengthened in two significant ways by treating transport with this degree of caution: it avoids overstating mechanistic certainty and offers a more solid basis for further consideration of crop performance, edible tissues, and food-system importance.

## 6. ROS-Centered Stress Signaling and Physiological Disruption

Across the plant MPs/NPs literature, one of the most consistent response patterns is disruption of redox homeostasis. Oxidative imbalance is a major pathway through which particle exposure is translated into physiological disruption, as evidenced by numerous reports of increased reactive oxygen species (ROS), altered antioxidant enzyme activity, lipid peroxidation, and related declines in chlorophyll content, photosynthetic efficiency, and plant growth [63,76,77,78,79]. This recurrent pattern is particularly significant in agricultural systems because it establishes a mechanistic connection between phenotypes that are crucial for agronomic performance and particle-associated stress at the plant interface.

However, ROS should not be used exclusively as a sign of damage. ROS may serve as an early signaling element that triggers antioxidant defenses, stress-responsive transcription, and physiological adaptation under moderate, brief, or lower-intensity exposure. ROS accumulation may surpass buffering capability under more severe, persistent, or chemically complex exposure, shifting toward membrane damage, pigment loss, reduced photosynthesis, and growth inhibition. This distinction helps explain why studies of broadly similar particle classes sometimes report weak or mixed responses, whereas others report clear suppression of root growth, biomass accumulation, or physiological performance. Therefore, the biological significance of ROS depends not only on whether it is induced but also on whether the induced state continues to be compensatory or develops into sustained harm.

This redox perspective is most instructive when interpreted alongside nutrient and ion regulation. Numerous studies have shown that oxidative imbalance is accompanied by disturbed membrane function, altered nutrient uptake, disrupted ion balance, and weaker root performance, all of which can affect carbon assimilation and biomass production [63,77,79]. This implies that ROS-related disruption in agriculture is not limited to biochemical stress responses. Reduced chlorophyll maintenance, decreased photosynthetic capability, poorer nutrient efficiency, and eventually weaker crop establishment are broader physiological effects that may develop from root-level stress. Because of this, ROS-centered stress is not so much an isolated endpoint as it is a regulatory and functional bottleneck.

The explanatory power of ROS also lies in its ability to connect mechanistic stress with phenotypic expression, which is another aspect. Redox imbalance can cause root growth inhibition, decreased shoot vigor, decreased photosynthetic performance, decreased biomass accumulation, and changes in metabolic allocation, either directly or through interactions with larger physiological networks. In this way, ROS-centered signaling offers a useful means of linking earlier framework stages, specifically, interface disruption and limited transport, with subsequent crop-level results. Additionally, it explains why visible growth penalties do not always show up right away: in some systems, biochemical stress can be identified before obvious agronomic impairment manifests, while in others, prolonged or compounded stress pushes the plant beyond a compensatory threshold and into quantifiable performance loss.

**Figure 4:**
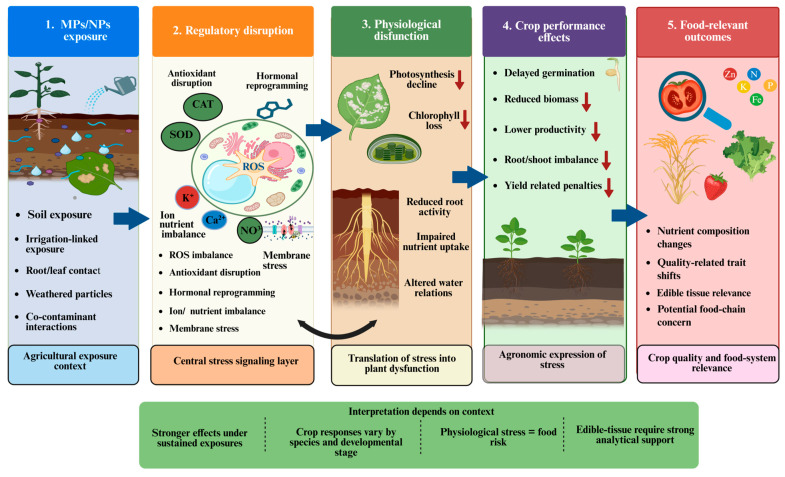
From physiological disruption to crops and food-relevant outcome.

This distinction is particularly relevant to the broader aims of the review. The effects of persistent oxidative disruption may extend beyond basic phytotoxicity to include characteristics that directly affect crop quality and food production, such as nutrient uptake, physiological function, and the composition of potentially edible organs. Therefore, in the particles-to-phenotypes framework, ROS-centered stress signaling plays a crucial role as the point at which particle-associated disruption starts to take on agronomic significance. The progression from ROS-centered physiological disturbance to agronomic and food-relevant outcomes is synthesized in Figure 4, while Table 3 summarizes the major response categories and their likely mechanistic links.

The response categories listed in Table 3 should not be interpreted as independent endpoints. In most crop systems, they are linked through overlapping processes, especially root–surface stress, oxidative imbalance, disturbed nutrient relations, and reduced photosynthetic performance. The table is intended to summarize the main routes through which particle exposure can progress from early physiological disturbance to agronomically and food-relevant outcomes.

## 7. Crop Quality, Edible Tissues, and Food-System Relevance

When phytotoxicity is no longer a suitable endpoint, the question of food-system relevance arises. In agricultural systems, the central question is not only whether MPs/NPs affect plant development, but whether they influence crop quality, edible tissues, and human exposure through food consumption. Although there are significant analytical and interpretive limitations that limit this method, current evidence indicates that it is possible and increasingly supported. Reviews focused on fruits and vegetables now conclude that micro- and nanoplastics have been reported in edible produce and may enter crop seedlings under controlled conditions. However, it is important to note that most of the evidence currently available comes from standardized particles and laboratory designs, which do not accurately reflect exposure in agricultural soils or irrigation-linked systems [10,16].

This distinction matters because the relevance of edible tissues is not the only factor that determines particle detection. Additionally, it relies on the location of the particles, how they were discovered, and whether the underlying evidence points to a real internal occurrence, surface retention, or external contamination. Early research by Conti et al. (2020) [90] reported micro- and nanoplastics in commonly consumed fruits and vegetables and estimated probable dietary intake from these foods, helping to establish the soil–plant–food pathways as a credible concern rather than a purely theoretical issue. However, subsequent evaluations have highlighted the continued high variability of analytical procedures, sample pretreatment, contamination control, and confirmation standards, which means that not all edible-tissue claims have the same weight in terms of evidence [16,90]. This heterogeneity is particularly significant in food-system research, where the distinction between external residues and internal tissue occurrence has direct implications for biological interpretation and exposure assessment.

Food relevance may also arise even when visible yield loss is limited. In addition to more well-known effects on biomass and growth, recent studies of crops and agroecosystems show that MPs/NPs can impact mineral nutrition, photosynthesis, metabolite accumulation, and toxicant transport within plant systems, potentially having an impact on food safety and quality [9,91]. From this perspective, the significance of MPs/NPs in crop production does not rely on whether particles accumulate in edible organs. Changes in co-contaminant behavior, physiological quality, or nutrient content may also impact on the value and safety of agricultural goods.

Literature is starting to shift from simple detection to exposure interpretation from the standpoint of the food chain. According to a recent predictive modeling study, humans may be exposed to micro- and nanoplastics through the consumption of fruits, vegetables, and grains, with vegetables accounting for the biggest predicted contribution [92]. Nevertheless, the study’s findings are better understood as a risk-oriented scenario rather than as a conclusive assessment of actual dietary exposure because it depended on assumptions about plant internalization and bioaccumulation [16,92]. Currently, the most defensible position is that edible crops represent a credible pathway linking contaminated soil and water to human exposure; however, before food-system risk can be confidently estimated, more field-based data, standardized analytical procedures, and a clearer differentiation between internal and external contamination are still required.

As a result, crop quality and edible tissue relevance should be regarded as one of the most essential, yet evidence-sensitive, aspects of the MPs/NPs literature. This is the point at which the current assessment most directly addresses broader issues about soil contamination, food quality, and public health. It is also the moment at which interpretative discipline counts most: overstating current evidence lowers credibility, but overlooking the soil–plant–food pathways overlook one of the key reasons microplastic contamination in agroecosystems has become such a significant environmental issue.

## 8. Evidence Gaps, Standardization Priorities, and Future Directions

Despite significant expansion in the MPs/NPs literature, various limitations continue to restrict the confidence with which current findings may be translated into agricultural and food-system implications. One of the most significant is exposure to realism. A large percentage of the existing evidence is still derived from simplified trials with clean particles, short exposure periods, hydroponic systems, or concentrations that are difficult to reconcile with actual crop settings. Reviews of agricultural soils and terrestrial plants consistently show that field-relevant interpretation necessitates a greater emphasis on weathered particles, mixed contamination pathways, and realistic soil- and irrigation-based exposure contexts, rather than relying solely on idealized laboratory designs. Without this change, mechanistic findings may stay true at the experimental level while providing only limited guidance for crop systems under real-world agricultural conditions.

A second restriction is uneven analytical rigor, notably in research on absorption, translocation, and edible-tissue occurrence. Recent reviews emphasize that conclusions in this domain are still extremely dependent on contamination control, washing techniques, labeling artifacts, detection limits, and whether polymer-specific confirmation is employed in conjunction with fluorescence- or imaging-based methods. This suggests that future success will be dependent not just on the generation of new datasets, but also on improving the evidentiary standard by distinguishing more explicitly between external association, supported internalization, and supported systemic translocation. This distinction is especially relevant when research reaches findings about crop quality, edible tissues, or dietary intake, because analytical overinterpretation can readily overtake what the data supports.

A third limitation is the persistently weak connection between mechanistic response and agronomic meaning. Many studies conclude with seedling growth tests, antioxidant measures, or isolated stress indicators, but few link these responses to nutritional status, photosynthetic function, productivity, edible-organ features, or crop-quality outcomes under real-world cultivation circumstances. As a result, the research frequently presents evidence of physiological disturbance without determining if that disturbance has any important implications for crop production, food quality, or human exposure. Future studies would be more informative if they combined environmental entry channels, interface behavior, transport evidence, and crop-relevant endpoints into a single experimental design rather than studying each variable separately.

These limitations point toward a more focused research agenda for the field. The use of weathered and environmentally relevant particles, more thorough polymer confirmation and contamination control, stronger integration of soil and irrigation exposure pathways, longer-term crop studies beyond early seedling stages, and a stronger focus on nutritional, metabolic, and edible-tissue traits should all be priorities. Studies should be planned to distinguish mechanical possibilities from agricultural relevance, which is equally vital. The question of whether MPs and NPs can affect plants should not be the only focus of future research; it should also include the environmental circumstances, the level of supporting evidence, and the implications for agricultural productivity and food systems.

Progress in these areas is essential if the field is to move beyond descriptive toxicity and toward robust inference for contaminated soils, irrigation-linked exposure pathways, and agricultural risk assessment. A more standardized, evidence-aware, and agriculture-centered approach will be necessary to determine when MPs/NPs represent a mechanistic curiosity, when they represent a credible agronomic stressor, and when they become a genuine concern for food-system safety.

## 9. Conclusions

Microplastics and nanoplastics are no longer peripheral contaminants in plant science; they are increasingly part of the environmental reality of modern agroecosystems. Their significance lies not only in their presence in contaminated soil and irrigation-linked crop systems, but also in the complexity of the pathways through which they influence plants. This review has argued that crop responses to MPs/NPs are best interpreted through a mechanistic chain linking environmental entry, interface behavior, barrier constraints, stress signaling, and phenotype expression at the level of germination, root function, photosynthesis, biomass accumulation, and quality-related traits.

A central conclusion is that plant effects cannot be inferred from polymer identity alone. The biological meaning of exposure depends on particle size, morphology, surface chemistry, weathering state, aggregation behavior, co-contaminant interactions, and, critically, the environmental matrix in which plant contact occurs. In many agricultural contexts, biologically meaningful effects may arise even when strong evidence for systemic internalization is limited, because particles can disrupt root–surface processes, rhizosphere functioning, nutrient dynamics, and physiological regulation without necessarily moving deeply into plant tissues. For this reason, a clear distinction among surface associations, supported internalization, and supported systemic translocation remains essential, particularly when conclusions are extended to edible tissues, crop quality, or food-chain relevance.

The review also shows that food-system relevance can no longer be treated as a secondary issue. Evidence from edible crops, fruits, and vegetables indicates that the soil–plant–food pathway is plausible and increasingly supported, but uneven analytical standards and limited field realism still constrain its interpretation. The most pressing need is therefore not simply a larger number of effect studies, but stronger studies that combine realistic agricultural exposure scenarios, rigorous confirmation methods, and crop-relevant endpoints, including edible tissues, nutritional quality, and longer-term physiological performance.

The field now needs to move beyond descriptive toxicity and toward evidence-aware, agriculture-centered inference. Progress will depend on integrating realistic exposure design, stronger analytical confirmation, and mechanistic interpretation with agronomic and food-system endpoints. Only through this shift can research on MPs/NPs provide a reliable basis for contamination assessment, crop management, and food-system risk evaluation in soil- and water-affected agricultural systems.

## Figures and Tables

**Table 1:** Key exposure pathways of microplastics and nanoplastics in agroecosystems and their likely crop relevance.

Exposure Pathway	Main Environmental Matrix	Typical Source/Material	First Likely Plant Contact	Main Likely Effect Route	Crop/Food-System Relevance	References
Mulch-film degradation	Agricultural soil	Weathered mulch fragments, film residues, secondary MPs	Root–soil interface	Surface retention, soil physical alteration, and rhizosphere disruption	May affect germination, root growth, soil quality, and crop productivity	[5,18]
Biosolids/sewage sludge application	Agricultural soil	Wastewater-derived MPs in sludge/biosolids	Root–soil interface	Soil loading, co-contaminant interactions, microbial and nutrient disturbance	Important for long-term soil accumulation and crop exposure	[7,12,45]
Compost/organic amendments	Agricultural soil	Plastic-contaminated compost, digestate, waste-derived organic amendments	Root–soil interface	Soil-mediated exposure, particle accumulation, altered soil–plant interactions	Relevant to organic input pathways and hidden contamination in food-production systems	[12]
Reclaimed or wastewater irrigation	Soil–water interface/irrigation water	MPs remaining in reclaimed water or transferred during irrigation	Root zone, soil pore water, sometimes leaf surface	Water-to-soil transfer, matrix-dependent root exposure, repeated loading	Directly links water contamination to crop systems and food-production landscapes	[43]
Atmospheric deposition	Air/leaf surface/soil surface	Airborne fibers and fine plastic particles	Leaf cuticle, canopy surface, soil surface	Foliar retention, external deposition, and secondary loading of soil	Relevant to edible-surface contamination and interpretation of crop exposure	[7]
Greenhouse/nursery plastic residues	Protected cultivation of soil/on-farm water and substrate environments	Greenhouse films, trays, tubing, nursery plastics	Root zone, nearby soil or substrate, irrigation runoff	Localized repeated release, enclosed-system accumulation	Especially relevant in horticultural systems with intensive plastic use	[46]
Fertilizer-associated inputs/coated fertilizers	Agricultural soil	Polymer-coated fertilizers and fertilizer-associated plastic residues	Root–soil interface	Localized release near active roots, mixed chemical–physical stress	Relevant to hidden field inputs and chronic soil loading	[47]
Surface runoff/flood redistribution	Soil–water interface	Plastic fragments are transported from nearby land, drainage, or waste sources	Soil surface, root zone	Secondary redistribution, mixed-source exposure	Important in fields influenced by runoff or episodic water movement	[9]

**Table 2:** Evidence strength for uptake, translocation, and edible-tissue claims in plant MPs/NPs studies.

Evidence Category/Method	What It Can Reasonably Support	Minimum Controls/Requirements	Main Limitations/Common Pitfalls	Interpretation Level	Key References
External surface observation (root cap, epidermis, leaf cuticle, fruit surface)	Surface association only	Careful washing, procedural blanks, and negative controls	External residues may be mistaken for uptake	Low	[10,14,15]
Fluorescence imaging alone	Suggestive localization only	Dye-only controls, autofluorescence controls, and validated washing	Dye leakage, plant autofluorescence, false-positive internal signal	Low to moderate	[10,15]
Confocal/microscopy with sectioning	Conditional evidence of internalization	Clean sectioning, contamination control, repeated imaging	Spatial signal may still be difficult to distinguish from preparation artifacts	Moderate	[72,73]
Polymer-specific spectroscopy (e.g., Raman/FTIR mapping)	Stronger support for polymer identity in tissues	Matrix-matched controls, blanks, spectral validation	Detection limits, signal interference from the plant matrix	Moderate to high	[68,74]
Bulk polymer confirmation (e.g., Py-GC/MS)	Stronger support for particle-derived polymer occurrence in tissues	Strict contamination control, digestion blanks, and recovery checks	Confirms polymer presence but not exact spatial localization	Moderate to high	[68]
Multi-method confirmation (imaging + polymer-specific method)	Supported internalization	Orthogonal confirmation, independent controls, clean workflow	Still requires a clear distinction between surface residues and internal compartments	High	[15]
Detection in distal non-edible tissues (e.g., shoots, leaves)	Supported translocation, if contamination is excluded	Spatial separation, replication, polymer confirmation	Distal detection alone is not proof of systemic movement without strong controls.	High, if validated	[75]
Detection in edible tissues	Supported edible-tissue occurrence only when internal vs. external contamination is clearly separated	Surface cleaning validation, orthogonal confirmation, contamination controls across handling and analysis	Postharvest contamination, surface deposition, and lab contamination can confound interpretation.	Highest required standard	[75]

Low-level evidence is useful for demonstrating contact or suggestive localization, but it is insufficient on its own for strong uptake claims. Moderate-level evidence provides more credible support for internal occurrence but remains limited without orthogonal confirmation. High-level evidence is appropriate for stronger inference when contamination control and polymer-specific confirmation are both robust.

**Table 3:** Main crop-relevant outcomes of MPs/NPs exposure and their likely mechanistic links.

Response Category	Typical Indicators	Likely Mechanistic Link	Agronomic Significance	Food-System Relevance	References
Germination and seedling establishment	Germination %, emergence rate, early shoot/root length	Surface adherence, impaired water uptake, and early oxidative stress	Poor stand establishment, weaker early vigor	Indirectly affects crop establishment rather than direct food risk	[80,81]
Root architecture and belowground performance	Root length, branching, root activity, absorptive surface	Root–surface stress, rhizosphere disruption, and altered soil physical and nutrient conditions	Reduced resource capture, weaker plant establishment, and resilience	Indirect but important, because root dysfunction can affect later yield and quality	[82,83]
Photosynthetic performance	Chlorophyll content, gas exchange, chloroplast damage	ROS imbalance, membrane stress, chlorophyll loss, nutrient disturbance	Reduced carbon assimilation and growth potential	Can influence edible-organ development and quality traits under sustained exposure	[9,71]
Biomass accumulation and productivity	Shoot/root biomass, plant height, yield-related traits	Integrated outcome of root impairment, oxidative stress, and reduced photosynthesis	Direct agronomic significance: yield and productivity penalties	Relevant to food production quantity	[9,24,84]
Nutrient uptake and ion balance	N, P, K, micronutrients, ion ratios, mineral status	Disturbed root uptake, membrane transport disruption, and altered soil nutrient availability	Lower nutrient efficiency, reduced crop performance	Relevant to nutritional quality and edible-organ composition	[85,86]
Metabolic and quality-related shifts	Phenolics, flavonoids, antioxidant metabolites, stress metabolites, quality markers	ROS-centered signaling, defense allocation, metabolic reprogramming	May occur even without large biomass loss	Directly relevant to crop quality and potential food-value changes	[87,88]
Edible tissue occurrence/edible-organ concern	Detection in fruits, vegetables, grains, and edible tissues	Conditional transport or external contamination requires the strongest analytical confirmation	Not an agronomic trait alone, but critical for interpretation	Highest relevance to food exposure and public health discussion	[16,89]

## Data Availability

No data was used for the research described in the article.
